# 样品制备方法对高效液相色谱-串联质谱分析人乳内源肽的影响

**DOI:** 10.3724/SP.J.1123.2020.08019

**Published:** 2021-05-08

**Authors:** Wenhao YU, Yang YU, Wendan WANG, Yitong LI, Ignatius M. SZETO, Yan JIN

**Affiliations:** ^1^.中国科学院大连化学物理研究所, 中国科学院分离分析重点实验室, 辽宁 大连 116023; ^1^. CAS Key Laboratory of Separation Science for Analytical Chemistry, Dalian Institute of Chemical and Physics, Chinese Academy of Sciences, Dalian 116023, China; ^2^.北京伊利科技发展有限责任公司, 北京 100070; ^2^. Beijing Yili Technology Development Co., Ltd., Beijing 100070, China

**Keywords:** 液相色谱, 串联质谱, 内源肽, 人乳, 提取方法, liquid chromatography (LC), tandem mass spectrometry (MS/MS), endogenous peptides, human milk, extraction method

## Abstract

人乳内源肽是乳蛋白在乳腺中被降解形成的具有生理功能的肽,是人乳的重要组成部分,研究人乳内源肽对于婴儿健康具有重要的意义。高效液相色谱-串联质谱(LC-MS/MS)联用技术的应用,促使人乳内源肽的研究取得了突破性的进展。人乳中内源肽含量低、干扰组分多,样品制备方法是影响分析结果的关键步骤。为了研究样品制备方法对分析结果的影响,分别采用不变性超滤法(UF 1)、热变性超滤法(UF 2)、化学变性超滤法(UF 3)、三氯乙酸沉淀法(PCPN 1)、乙醇沉淀法(PCPN 2)、强疏水性碳介孔材料(highly ordered mesoporous carbon, OMC)富集法等6种方法从人乳中提取内源肽,利用LC-MS/MS研究样品制备方法对人乳内源肽分析结果的影响。结果表明,UF 1和UF 2法制备的样品中可鉴定到的肽段数目分别为1161±8条和1017±91条,两种方法制备的样品中肽序列的重合率大于70%, UF 1在所有方法中鉴定到的肽的数目最多。UF 3法制备的样品所能鉴定到的肽段数目最少,仅为366±18条。PCPN 1和PCPN 2两种沉淀法制备样品中的内源肽分别为779±69和876±55条,但内源肽差异较大,仅有约50%肽段序列重合。OMC法制备样品中肽的数目为549±151条,与其他方法相比,虽然鉴定的肽数量上没有优势,但该方法制备的样品中肽在等电点(pI)和亲水性平均系数(GRAVY)等性质上没有偏倚,说明该法可用于制备特定人乳内源肽。6种方法制备的样品中鉴定到来源于*β*-酪蛋白、免疫球蛋白受体、骨桥蛋白、*α*_S1_-酪蛋白、*κ*-酪蛋白和胆盐激活脂肪酶的肽,并且源于以上蛋白质的肽段总数在该样品中均超过88%,说明6种方法制备的样品都可以满足鉴定一般人乳内源肽的需求。UF 2、UF 3和OMC法制备的样品中鉴定到源于乳铁蛋白的内源肽的数目分别为21、38和19条,内源肽在乳铁蛋白上的覆盖率分别为14%、16%和19%,而文献常用的PCPN 1法制备的样品则会丢失此类内源肽。综上,UF 2法制备的样品不仅肽段数量多、母体蛋白质种类丰富,还可鉴定到源于乳铁蛋白的肽,可作为人乳内源肽组学研究中的首选方法。

## Abstract (en)

Hundreds of endogenous peptides were released from milk proteins within the human mammary gland and some of them possess a variety of bioactive functions. Thus, it is important to investigate human milk endogenous peptides for infant health. Peptidomics based on liquid chromatography-tandem mass spectrometry (LC-MS/MS) has been used to investigate human milk endogenous peptides. Extraction of endogenous peptides from human milk is an essential and key procedure for analyzing human milk peptides using LC-MS/MS. This study aimed to compare methods for extracting endogenous peptides from human milk using LC-MS/MS. Ultrafiltration methods including that not involving denaturation (UF 1), that involving heat denaturation (UF 2), and that involving chemical denaturation (UF 3), precipitation methods using trichloroacetic acid (PCPN 1) and alcohol (PCPN 2), and an enrichment method using highly ordered mesoporous carbon (OMC) were used to extract endogenous peptides from human milk. Extracted endogenous peptides were then analyzed using LC-MS/MS. The samples extracted using UF 1 and UF 2 comprised 1161±8 and 1017±91 endogenous peptides, respectively. More than 70% peptide sequences in each sample extracted using UF 1 and UF 2 overlapped. The results revealed that endogenous peptides extracted using UF 1 and UF 2 showed similar characteristics. UF 1 yielded the highest number of peptides, whereas UF 3 extracted the least number of peptides at 366±18. The number of endogenous peptides extracted using PCPN 1 and PCPN 2 were 779±69 and 876±55, respectively. However, their characteristics were quite different, and only about 50% peptide sequences overlapped. The number of peptides extracted using OMC (549±151) was not remarkable compared with that using other methods. However, the isoelectric point (pI) and grand average of hydropathicity (GRAVY) of the peptides extracted using OMC were different from those extracted using other methods. This method presented no selectivity for the endogenous peptides with different pI and GRAVY and may be used to extract unique peptides from human milk. A total of 205 peptides were commonly identified in the samples using each of the six methods. The percent of shared peptides across the six samples ranged from 13% to 23%. The number of unique peptides in the samples extracted using UF 1 and UF 2 (226 and 228, respectively) were the highest among those extracted using the six methods. The results showed that all six methods could be used to extract endogenous peptides from these high-abundance precursor proteins. A total of 21, 38, and 19 peptides were extracted from lactotransferrin using UF 2, UF 3, and OMC, respectively, and the coverage rates of these peptides in lactotransferrin were 14%, 16%, and 19%, respectively. These three methods could extract the endogenous peptides from lactotransferrin in human milk, but PCPN 1 that has been commonly used in previous studies could not. The peptides from *β*-casein, polymeric immunoglobulin receptor, osteopontia, *α*_S1_-casein, *κ*-casein, and bile salt-activated lipase were identified in all samples extracted using the six methods. Moreover, these precursor proteins contributed 88% peptides in the samples extracted using the six methods. In conclusion, UF 1 and UF 2 were efficient procedures for extracting endogenous peptides from human milk. In addition, UF 2 could extract peptides from lactotransferrin, which is the optimum choice for extracting endogenous peptides from human milk. Additionally, the OMC enrichment method can be used to enrich and extract specific endogenous peptides from human milk. This study systematically compared the sample preparation methods commonly used in human milk endogenous peptidomics in recent years. The results provide strong support for uniform and standardized sample preparation methods. An ultrafiltration method without denaturation, which is more advantageous than the currently commonly used trichloroacetic acid precipitation method, was also established to prepare human milk endogenous peptide samples. In combination with OMC, this method can help in a more comprehensive and in-depth understanding of the endogenous peptidome of human milk.

人乳是婴儿最早的食物,不仅为婴儿提供生长发育所需的营养,而且为人类提供构建最初免疫系统的活性因子。蛋白质是人乳中最重要的营养成分之一,大部分蛋白质随乳汁分泌后被婴儿食用并消化吸收,但是还有部分蛋白质在乳腺中就被乳汁中的蛋白酶降解并泌出体外,形成了人乳的重要组分内源肽。人乳内源肽与婴儿生长发育密切相关。研究发现,母亲自身机能可根据婴儿健康调节内源肽的产生^[1]^,早产儿的自身消化系统还不完善不能消化大量蛋白质,需要更多内源肽满足早产儿的营养需求,因此肽的浓度和丰度等指标在早产的人乳样品中显著高于足月产的人乳样品^[2]^。对人乳内源肽功能研究发现,人乳内源肽具有抗菌^[3,4]^、免疫调节和调节肠道菌群^[5]^等多种生理功能^[6]^,这些功能对于婴儿免疫系统建立具有重要作用。因此,研究人乳内源肽对于婴儿的健康、成长发育以及婴儿配方奶粉的研发具有重要意义。

基于LC-MS/MS的肽组学技术具有高效、高灵敏度的特点,在对人体血清^[7,8]^、唾液^[9]^等组织的肽组学研究中发挥重要作用,肽组学技术的应用也使得人乳内源肽的研究取得突破性进展^[10]^。Dallas等^[2]^利用肽组学技术揭示了人乳内源肽在乳腺内形成的机制。Holton等^[11]^利用肽组学技术研究人乳内源肽在消化过程中的变化。Gan等^[12]^利用肽组学技术研究了pH对人乳中蛋白质和蛋白酶体系的影响。肽组学技术的应用揭示了人乳内源肽的产生、消化过程以及与婴儿生长发育的关系。但是内源肽在人乳中的含量非常低,高丰度蛋白质及其他成分干扰内源肽的分析检测,因此内源肽的分离制备是研究人乳内源肽必不可少的关键步骤。目前常用的肽制备方法有三氯乙酸(trichloroacetic acid solution, TCA)沉淀法^[2]^、离心超滤法^[13,14]^、有机溶剂沉淀法^[15,16,17]^,以及利用强疏水性碳介孔材料(highly ordered mesoporous carbon, OMC)富集法^[18]^。目前文献报道的人乳内源肽研究中大多采用TCA沉淀法制备内源肽^[12,19]^,因此关于样品制备方法对于人乳内源肽分析结果影响的研究非常少见^[20]^。为了比较不同制备方法对人乳内源肽的影响,本研究分别采用不变性、加热变性和化学变性后超滤的超滤法、有机溶剂沉淀和酸沉淀的沉淀法以及OMC材料富集法共6种方法制备人乳内源肽,利用LC-MS/MS研究样品制备方法对人乳内源肽分析结果的影响。

## 1 实验部分

### 1.1 仪器、材料、试剂与样品

LC-MS/MS系统由Agilent 1100 HPLC系统和LTQ-Orbitrap Velos质谱仪(配有纳升电喷雾离子源及Xcalibur 2.1数据处理系统)组成。SpeedVac真空冷冻干燥机,美国Thermo公司。Milli-Q超纯水一体机和超滤管(10 kDa, 0.5 mL)购自美国Millipore公司。Allegra 64R台式高速冷冻离心机,美国Beckman公司。

石英毛细管购自美国Polymicro Technologies公司。Waters Oasis HLB固相萃取小柱购自美国Waters公司。OMC材料为中国科学院分离分析重点实验室自制。

甲酸(formic acid, FA,色谱纯)购自美国Fluka公司;乙腈(acetonitrile, ACN,色谱纯)购自德国Merck公司。甲醇(色谱纯)购自美国Thermo公司。三氟乙酸(trifluoroacetic acid, TFA)、三氯乙酸(trichloroacetic acid solution, TCA)、脲、二硫苏糖醇(dithiothreitol, DTT)等试剂购自美国Sigma公司。其他有机溶剂购自天津市科密欧化学试剂有限公司。

人乳样品由北京伊利科技发展有限责任公司提供,样品采自45位居住于哈尔滨、青岛、呼和浩特3个城市分娩后3~17天的母亲,乳母年龄为23~39周岁。样品采集后冷冻于-80 ℃,本研究所使用的样品是45份样品的混合物。

### 1.2 人乳内源肽的制备方法

采用了超滤法、沉淀法和OMC材料富集法3种方法制备人乳内源肽。超滤法中分别考察了不变性(UF 1)、热变性(UF 2)和化学变性(UF 3)3种变性方式对内源肽的影响;沉淀法中分别考察了酸沉淀(PCPN 1)和有机溶剂沉淀(PCPN 2)两种沉淀方式的影响。内源肽制备方法和编号见表1。

**表 1:** 人乳内源肽的制备方法

No.	Method
UF 1	10 kDa ultrafiltration
UF 2	heat denaturation, 10 kDa ultrafiltration
UF 3	chemical denaturation, 10 kDa ultrafiltration
PCPN 1	trichloroacetic acid precipitation
PCPN 2	alcohol precipitation
OMC	highly ordered mesoporous carbon enrichment

1.2.1 超滤法

不变性:取200 μL人乳用去离子水稀释5倍混合均匀。热变性:参考Wang等^[7]^的方法,取200 μL人乳用去离子水稀释5倍混合均匀,稀释液在水浴95 ℃变性5 min。化学变性:参考Wan等^[21]^的方法,取200 μL人乳加入800 μL蛋白质变性液(7 mol/L脲、2 mol/L硫脲、20 mmol/L DTT),混合均匀变性30 min。不变性、热变性和化学变性后的样品分别用截留相对分子质量10 kDa的超滤管在14000 g、20 ℃条件下超滤30 min,收集超滤液分别得到UF 1、UF 2和UF 3法制备的样品,超滤液冷冻干燥后进行后续处理。

1.2.2 沉淀法

酸沉淀法:参考Dallas等的方法^[2,22]^,取200 μL人乳与200 g/L TCA以体积比1∶1混合,充分混匀后在14000 g、4 ℃条件下离心20 min,收集上清液为PCPN 1样品。有机溶剂沉淀法:取200 μL人乳与800 μL有机溶剂(50%乙醇+50%丙酮+0.1%醋酸)混合,充分混匀后于-20 ℃过夜沉淀,在14000 g、4 ℃条件下离心20 min,收集上清液为PCPN 2样品。酸沉淀法和有机溶剂沉淀法获得的上清液冷冻干燥后进行后续处理。

1.2.3 OMC富集法

取20 μL人乳,加入120 μL 0.1%(v/v)TFA水溶液,95 ℃保温5 min后加入30 μL质量浓度为30 g/L的OMC材料,振荡孵育30 min,在20000 g、20 ℃离心3 min,弃去上清液,加入100 μL 0.1%(v/v) TFA水溶液混匀后离心弃去上清液,以上操作重复两次。加入100 μL 80%(v/v)ACN/0.1%(v/v)TFA水溶液混匀后离心收集上清液,以上操作重复两次,合并收集到的上清液,即为OMC样品,冷冻干燥后用于后续处理。

### 1.3 样品脱盐处理

经过1.2节不同方法处理的样品分别用SPE柱除盐,步骤如下:SPE柱用1.5 mL甲醇活化后加入1.5 mL 0.1%(v/v)TFA水溶液平衡,将样品以0.5 mL 0.1%(v/v) TFA水溶液溶解后加入SPE柱中,以1.5 mL 80%(v/v) ACN/0.1%(v/v) TFA-H_2_O溶液进行洗脱,收集洗脱液冷冻干燥保存于-80 ℃。

### 1.4 LC-MS/MS分析

脱盐后的样品以0.1%(v/v)甲酸水溶液溶解并制备成质量浓度为0.2 g/L的溶液,上样量为8 μL,加载到15 cm毛细管分析柱(内径为180 μm,填充C_18_ AQ填料)中进行液相色谱-质谱分析。流动相A为含0.1%甲酸的水溶液,流动相B为含0.1%甲酸的98%乙腈溶液。梯度洗脱程序如下:0~80 min, 5%B~25%B; 80~95 min, 25%B~35%B; 95~97 min, 35%B~90%B; 97~107 min, 90%B; 107~109 min, 90%B~0%B; 109~126 min, 0%B;流速70 μL/min。在Orbitrap质量分析仪中以60000的分辨率获得了完整的质谱扫描图(*m/z* 400~2000)。对其中最强的15个离子进行离子碰撞解离(CID),再进行MS/MS扫描,扫描范围在*m/z* 400~2000。动态排除功能设置如下:重复2;持续时间30 s;排除持续时间为60 s。系统控制和数据收集由Xcalibur软件进行。每个样品进行3次质谱分析。

### 1.5 数据检索

Xcalibur采集的*. RAW文件用Thermo Proteome Discoverer (v1.4)转换成*. MGF格式,之后用Mascot 2.5.1软件在人蛋白质数据库(Human spains,蛋白质数目为22427, http://www.uniprot.org/)中进行检索。搜库参数如下:不设置酶切、最大漏切数和固定修饰,可变修饰设置为甲硫氨酸的氧化(+15.9949 Da)。母离子的质量容忍偏差为20 ppm,碎片离子为0.8 Da。导出肽段时控制假阳性率(FDR)<1%,对导出结果Score > 20的肽段视为有效数据进行分析。

## 2 结果与讨论

### 2.1 LC-MS/MS分析内源肽的基峰色谱图

将人乳样品分别经过表1所列的方法处理后进行LC-MS/MS分析,图1为在相同分析条件下不同方法制备的人乳内源肽的基峰色谱图。UF 1、UF 2、UF 3与OMC法制备的样品的色谱峰在10~100 min的时间内均有分布,说明超滤法和OMC法制备的样品中肽段极性较宽泛。经PCPN 1和PCPN 2两种沉淀法制备的样品显示出较大差别,其中PCPN 1法制备的样品谱峰主要集中在10~70 min,说明该样品中亲水性肽较多;PCPN 2法制备的样品的谱峰主要集中在50~100 min,说明该样品中疏水性肽段较多。

**图 1:**
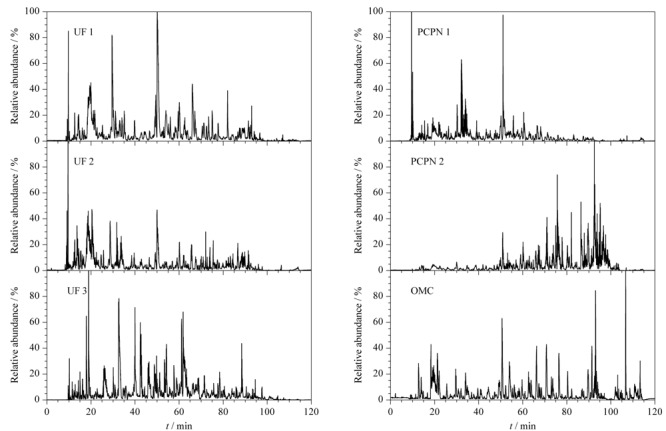
不同方法制备的人乳内源肽的LC-MS/MS基峰色谱图

### 2.2 不同方法制备的人乳内源肽及其母体蛋白质

每个样品经过3次平行实验,在3次实验结果中均被鉴定到的肽视为共有肽段;3次结果鉴定到的所有肽段合并删除重复肽段后得到的肽段为该方法的总肽段鉴定结果;共有肽段数占总肽段数的百分比为共有肽段百分比。母体蛋白质为释放内源肽的蛋白质,其数据处理与肽的处理方法一致。

**表 2:** LC-MS/MS鉴定的不同方法制备的人乳内源肽及其母体蛋白质数目(*n*=3)

Method	Peptide						Protein				
	Number		RSD/%	Number overlapped	Percent overlapped/%		Number	RSD/%		Number overlapped	Percent overlapped/%
UF 1	1161±	8	0.65	795	51.49		27±1	2.17		22	68.75
UF 2	1017±	91	8.98	573	38.48		26±2	7.91		16	42.11
UF 3	366±	18	4.89	222	42.37		11±2	21.65		7	46.67
PCPN 1	779±	69	8.84	484	44.00		15±2	13.58		11	52.38
PCPN 2	876±	55	6.29	542	44.28		18±3	14.70		13	59.09
OMC	549±	151	27.55	280	31.82		14±3	21.12		8	44.44

不同方法制备的样品经LC-MS/MS分析,所鉴定到的肽及其母体蛋白质的数目结果见表2。由表2可见,不同方法制备的样品中所能鉴定到的肽段数目存在很大差别,其中UF 1法和UF 2法制备的样品鉴定到的肽段数目最多,分别为1161±8条和1017±91条;化学变性法UF 3鉴定的肽段数目最少,只有366±18条。两种沉淀法PCPN 1和PCPN 2鉴定的肽段数目非常接近,分别为779±69和876±55条。OMC法制备的样品中鉴定到的肽段数目为549±151条。样品中鉴定的内源肽的母体蛋白质数目与内源肽数目表现出类似的规律。

共有肽段百分比和肽段数目的相对标准偏差(RSD)反映了样品数据的稳定性。UF 1法制备的样品中共有肽段百分比最高为51.49%, OMC法制备的样品中共有肽段百分比最低仅为31.82%,其他4种方法制备的样品百分比比较接近,为38.48%~44.28%。同一样品3次重复实验的结果中肽段数目的RSD顺序依次为:UF 1法制备的样品中鉴定到肽段数目的RSD最低为0.65%, OMC法制备的样品中鉴定到肽段数目的RSD最高为27.55%,其他3种方法制备的样品中鉴定到肽段数目的RSD均小于10%。每种样品中鉴定到的母体蛋白质中共有蛋白质的百分比与共有肽段的百分比显示出相似的变化规律。

以上结果说明,UF 1法制备的样品所能鉴定到肽段数目最多,数据的稳定性最好;UF 2法制备的样品所能鉴定到的肽段数目与UF 1法制备的样品的数目最接近,但稳定性较差;UF 3法制备的样品所能鉴定到的肽段数目最少;PCPN 1法制备的样品和PCPN 2法制备的样品所能鉴定到的肽段数目和数据稳定性都接近;OMC法制备的样品的数据稳定性最差。目前人乳内源肽组学研究的文献中^[2,22,23]^常采用PCPN 1方法制备的内源肽,从以上结果来看该方法制备的样品在鉴定肽段数目、数据稳定性等方面均不占优势。

### 2.3 不同方法制备的人乳内源肽的性质

将LC-MS/MS鉴定到的内源肽的长度、等电点(isoelectric point, pI)及亲水性平均系数(grand average of hydropathicity, GRAVY)等性质进行计算,不同性质的肽段占比的统计结果见图2。6种方法制备的样品所鉴定到的肽的氨基酸数目为6~45,其中11~20个氨基酸的肽占比最大,其次是21~30个氨基酸的肽和氨基酸数≤10个的肽,不同方法制备的肽在氨基酸数目分布方面没有明显差别。6种方法制备的人乳内源肽的等电点范围为2.00~13.00,除了OMC法外其他5种方法制备的样品的肽的等电点分布非常类似,即pI为4.01~5.00的肽占比最大。而OMC法制备的肽在不同pI间的百分比非常接近,说明OMC法富集内源肽时对pI没有偏倚。6种方法制备的肽的GRAVY在-3.00~2.00的范围,所有的样品中GRAVY在-0.99~0.00内的肽占比都最大,6种方法制备的样品中GRAVY为0.01~1的肽占比OMC法最高。以上结果说明,除OMC法外其他5种方法制备的人乳内源肽的物理性质差别不大,OMC法制备的人乳内源肽的pI和GRAVY均显示与其他5种方法存在差别,说明OMC法制备的样品结果显示一定的特异性。

**图 2:**
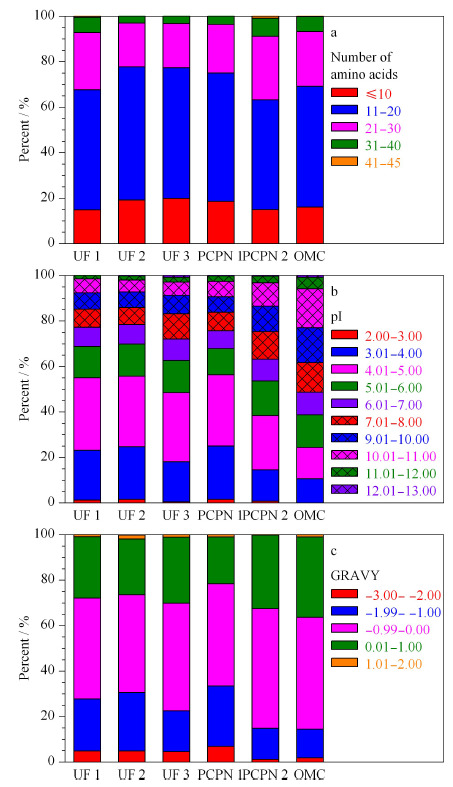
不同方法制备的人乳内源肽的性质

### 2.4 不同方法制备的人乳内源肽的差异

为了比较不同方法制备的样品中所鉴定到的肽段的差异,对同一肽段在不同样品中鉴定到的次数进行统计,结果见图3。图3中的6个色块分别代表同一肽段在6种方法制备的样品被鉴定的次数,例如6表示同一个肽段在6个样品中均被鉴定到,5表示同一肽段在5个样品中鉴定到,以此类推。6个样品中都能鉴定到的共有肽段有205条,分别是来源于*β*-酪蛋白的171条、*α*_s1_-酪蛋白的14条、免疫球蛋白受体的9条、骨桥蛋白的6条和*κ*-酪蛋白的5条。共有肽段数占各样品总肽段数的百分比分别为13%(UF 1)、14%(UF 2)、39%(UF 3)、19%(PCPN 1)、17%(PCPN 2)和23%(OMC)。虽然UF 3法和OMC法制备的样品中鉴定到的总肽段数目较少,但共有肽段占比较高。图3中1表示某个样品中所能鉴定到的独有肽段,6个样品独有肽段占比在11%~16%的范围内,UF 1法和UF 2法制备的样品中鉴定到的独有肽段数目最多分别为226和228条。UF 1和UF 2制备的样品中不仅肽的总数最高而且独有肽段数目最多。

**图 3:**
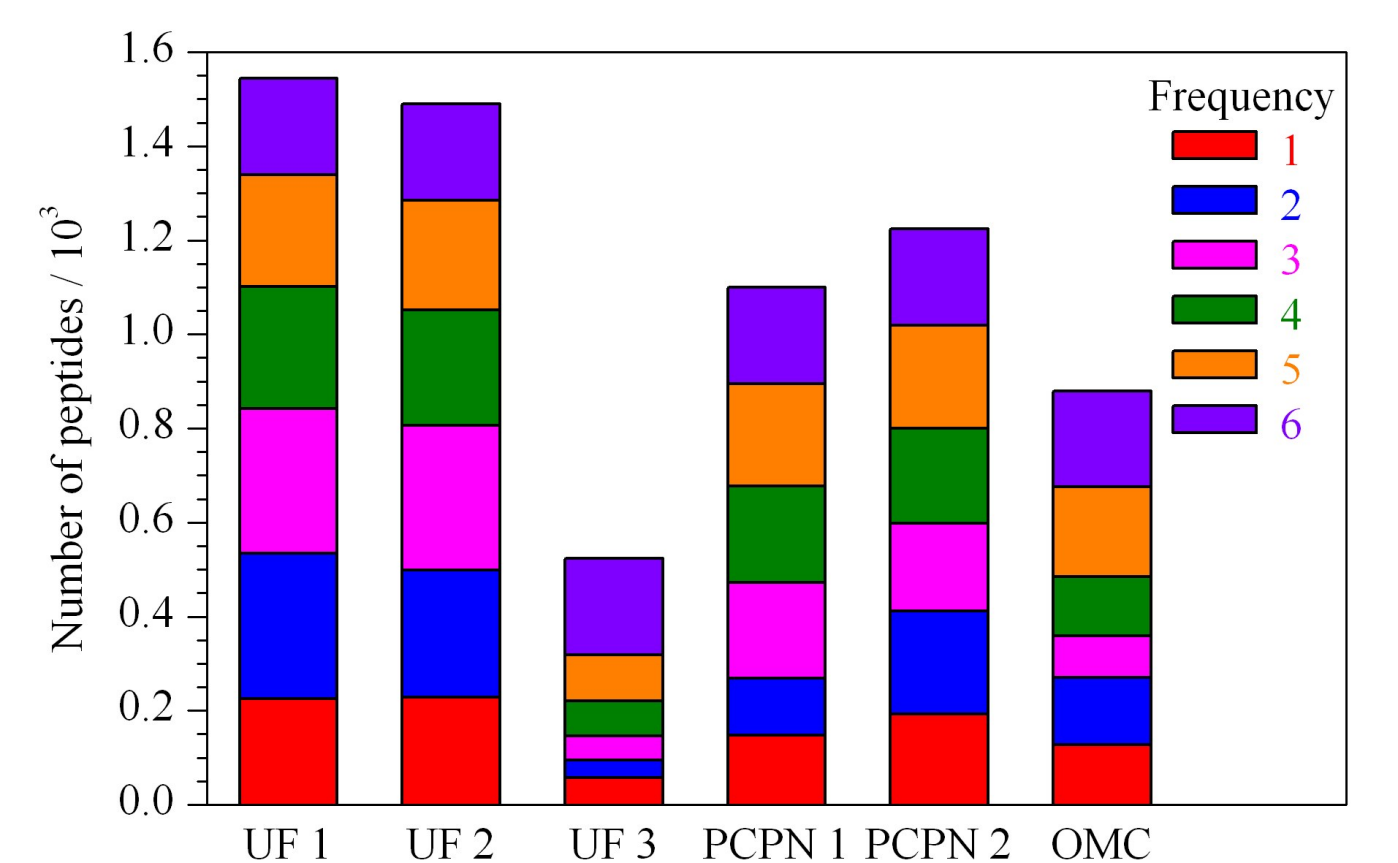
不同方法制备的样品中鉴定到的肽段的个数及频次

为了比较不变性、热变性和化学变性对超滤结果的影响,UF 1、UF 2和UF 3法制备的样品中鉴定到的总肽段结果的维恩图见图4a。3种超滤样品的共有肽段数目占其总肽段数目的百分比分别为68%、71%和83%,说明3种方法均能鉴定到人乳中大部分的内源肽,虽然化学变性的UF 3能鉴定到的肽段数目少,但该样品中83%的肽和另外两个样品中鉴定到的相同。UF 1和UF 2中所鉴定到的肽段的数目接近,两种方法制备的样品中共有肽段分别占其鉴定肽段的百分比为70%和73%,说明两种方法制备的肽非常接近,变性与否对所制备的肽影响不大,这个结果与Dallas证明不变性和热变性对三氯乙酸沉淀制备人乳内源肽没有影响的结论一致^[22]^。两种沉淀法PCPN 1和PCPN 2制备的样品的总肽段数目的维恩图见图4b,两种方法共有肽段数目占其总肽段数目的百分比分别是54%和49%,说明不同沉淀方法所制备的人乳内源肽差异较大。

**图 4:**
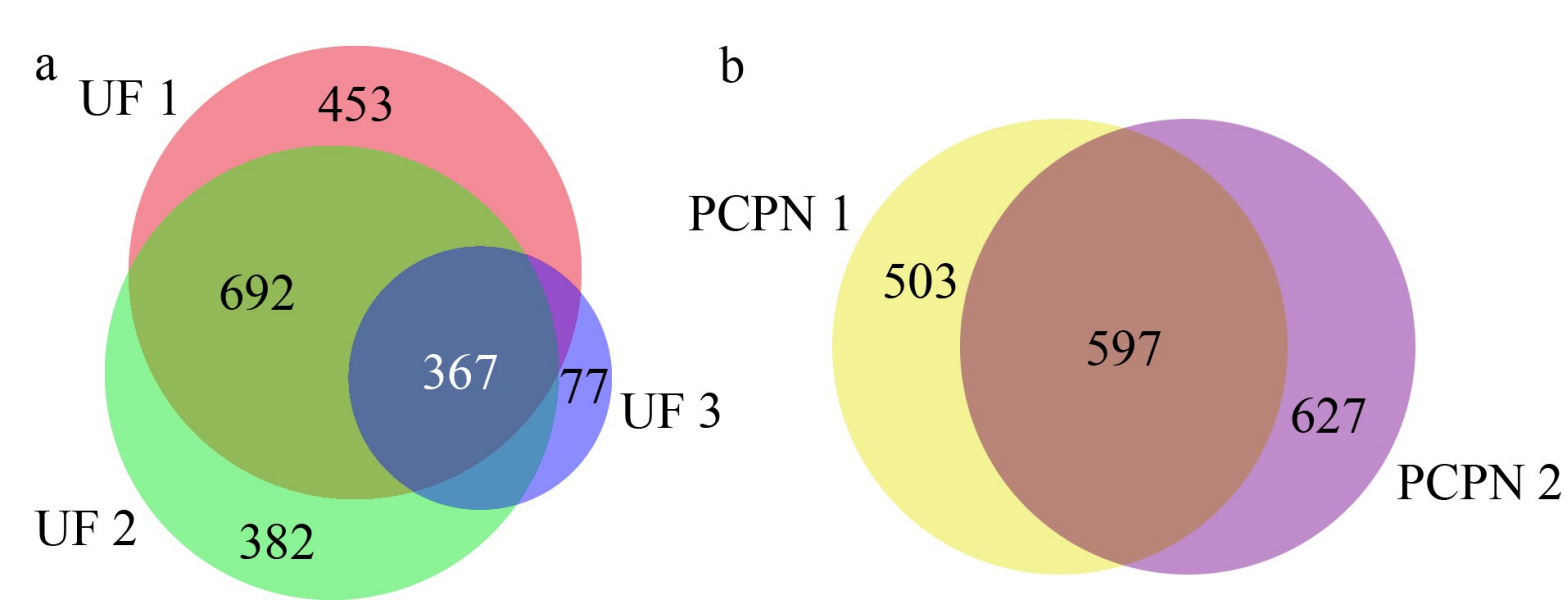
(a)超滤法和(b)沉淀法制备的样品中人乳内源肽的维恩图

### 2.5 人乳内源肽的母体蛋白质

不同方法制备的样品中释放内源肽数目最多的前十个母体蛋白质列于图5。6种方法制备的样品中均能鉴定到的母体蛋白质有:*β*-酪蛋白,免疫球蛋白受体、骨桥蛋白、*α*_S1_-酪蛋白、*κ*-酪蛋白和胆盐激活脂肪酶,来源于共有的母体蛋白质的肽段数目在该样品中占比均超过88%,说明6种方法制备的样品都可以满足鉴定一般人乳内源性多肽的要求。

**图 5:**
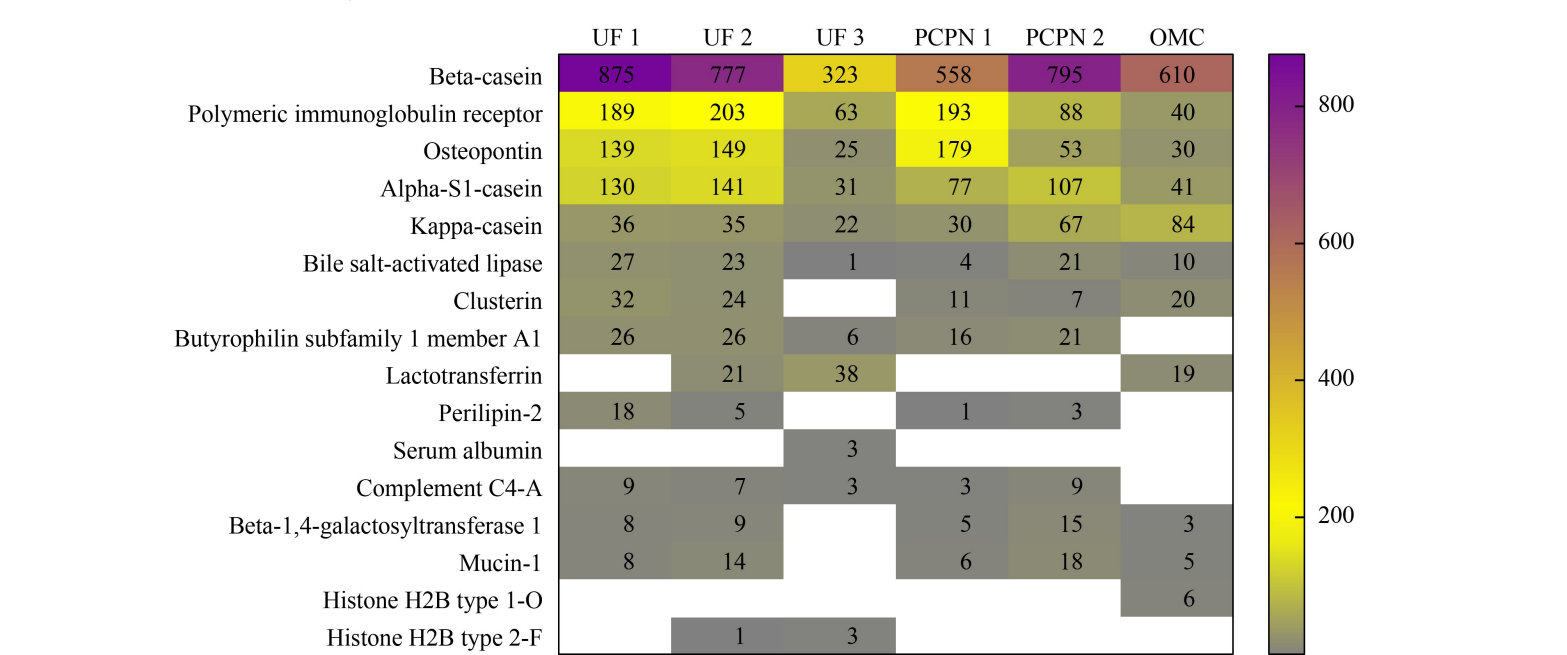
不同方法制备的样品中释放肽段数目最多的前10个母体蛋白质

乳铁蛋白是人乳中重要的糖蛋白,对于婴儿胃肠系统具有重要的作用,已证实乳铁蛋白释放的肽具有抗菌等作用,因此检测人乳中的乳铁蛋白及其肽段对于婴儿健康的研究非常重要。现有的人乳内源肽的研究采用三氯乙酸沉淀法处理样品,报道的结果中均未检测到来源于乳铁蛋白的内源性肽段^[22,23]^。由图5可见,UF 2、UF 3和OMC法制备的样品中均鉴定到乳铁蛋白释放的内源肽,分别为21、38和19条,而其他3种方法制备的样品未检测到源于乳铁蛋白的内源肽。说明不同样品制备方法可能导致源于乳铁蛋白的特异性肽段丢失。

图6展示了所鉴定到的内源肽在母体蛋白质上的覆盖率,*β*-酪蛋白释放的内源肽数目最多,肽段在*β*-酪蛋白上的覆盖率也最高,这与*β*-酪蛋白结构疏松、亲水性强的特点相关^[24]^。免疫球蛋白受体蛋白释放的内源肽数目较多,但蛋白质覆盖率相对较低,说明免疫球蛋白受体蛋白释放内源肽的区域相对较集中。所有样品中内源肽在骨桥蛋白和*α*_s1_-酪蛋白的覆盖率较高,UF 1、UF 2和PCPN 1法制备的样品中内源肽在骨桥蛋白和*α*_s1_-酪蛋白的覆盖率均超过70%。UF 2、UF 3和OMC法制备的样品中检测到来源于乳铁蛋白的内源肽在乳铁蛋白上的覆盖率分别为14%、16%和19%,说明源于乳铁蛋白的内源肽不是偶然检测到的,而是以上3种制备方法有利于这类肽的富集。OMC法制备的样品中来源于*κ*-酪蛋白的肽段数目最多为84条,蛋白质覆盖率也最高为55%;该样品也是唯一鉴定到6条来源于组蛋白H2B1-O型的内源肽,蛋白质覆盖率为41%。以上结果说明,不同的样品制备方法直接影响特异性肽的富集,可根据研究目的选择合适的制备方法。

**图 6:**
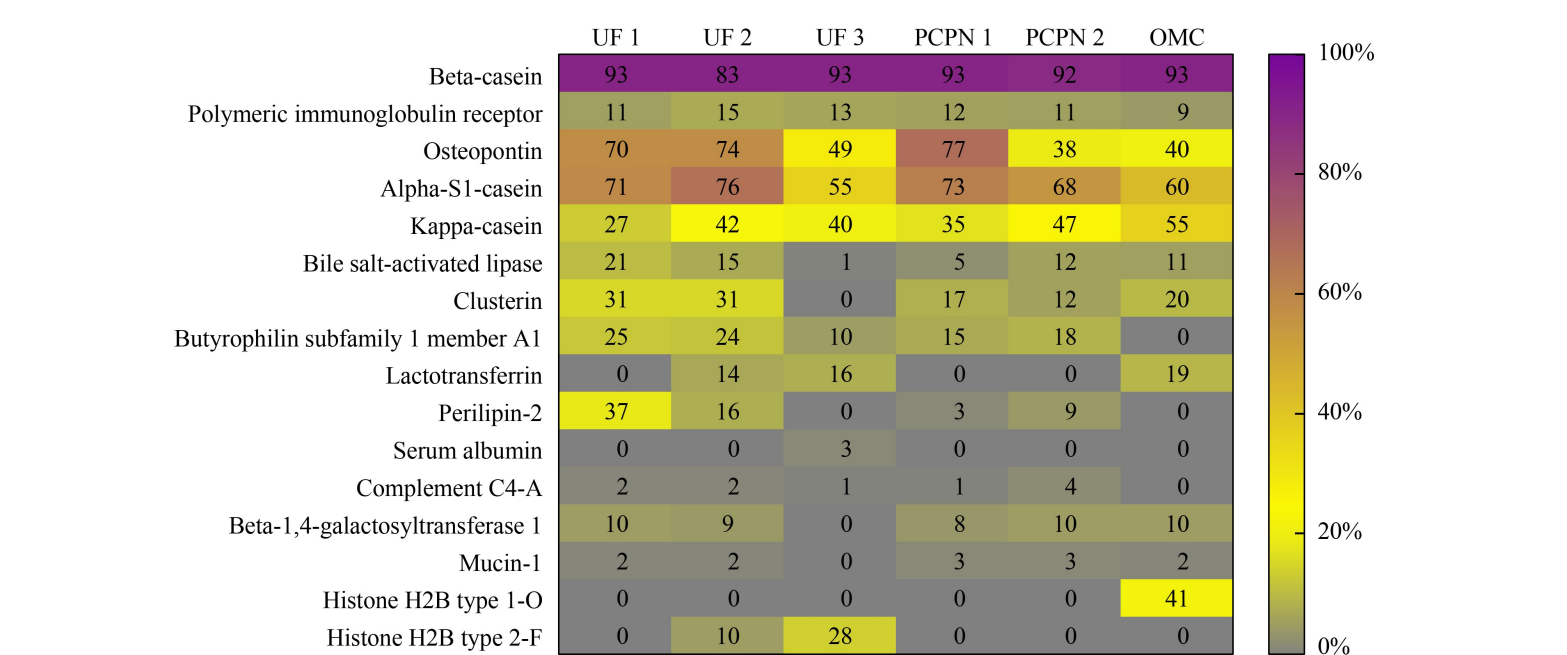
内源肽在母体蛋白质上的覆盖率

## 3 结论

人乳成分复杂多变且样品珍贵,样品制备方法对人乳内源肽研究有决定性的影响。本研究系统比较了不同样品制备方法对于LC-MS/MS分析人乳内源肽的影响,结果表明热变性超滤法制备的样品中内源肽的数目多,可富集到源于乳铁蛋白的内源肽,是较理想的制备人乳内源肽的方法。本文的研究结果表明人乳中存在对于婴儿健康重要的源于乳铁蛋白的内源肽,文献常用的样品制备方法导致这类内源肽丢失而未被检测到。因此,统一和规范样品制备方法对于研究结果的可比性具有重要的意义,本研究可为人乳内源肽的样品制备方法的选择提供借鉴。
